# Supplementary material for: The limits of nudging: Results of a randomized trial of text messages to promote home-based caregiving and reduce perceptions of stigma for COVID-19 patients in Kenyan informal settlements
Source: PLoS One. 2024 Aug 29;19(8):e0305206. doi: 10.1371/journal.pone.0305206 (PMC11361586; doi:10.1371/journal.pone.0305206)
Supplement: Attachment — Submitted filename: Revise_Resubmit_8.21.23.docx [file pone.0305206.s004.docx]

. Please ensure that your manuscript meets PLOS ONE's style requirements, including those for file naming. The PLOS ONE style templates can be found at 
<https://journals.plos.org/plosone/s/file?id=wjVg/PLOSOne_formatting_sample_main_body.pdf> and 
<https://journals.plos.org/plosone/s/file?id=ba62/PLOSOne_formatting_sample_title_authors_affiliations.pdf>

2. Please include a complete copy of PLOS’ questionnaire on inclusivity in global research in your revised manuscript. Our policy for research in this area aims to improve transparency in the reporting of research performed outside of researchers’ own country or community. The policy applies to researchers who have travelled to a different country to conduct research, research with Indigenous populations or their lands, and research on cultural artefacts. The questionnaire can also be requested at the journal’s discretion for any other submissions, even if these conditions are not met.  Please find more information on the policy and a link to download a blank copy of the questionnaire here: <https://journals.plos.org/plosone/s/best-practices-in-research-reporting>. Please upload a completed version of your questionnaire as Supporting Information when you resubmit your manuscript.

We have added this questionnaire as supporting information.

3. We note that the grant information you provided in the ‘Funding Information’ and ‘Financial Disclosure’ sections do not match.

When you resubmit, please ensure that you provide the correct grant numbers for the awards you received for your study in the ‘Funding Information’ section.

4. Thank you for stating the following financial disclosure: 
"The funders had no role in study design, data collection and analysis, decision to publish, or preparation of the manuscript."

At this time, please address the following queries:

a) Please clarify the sources of funding (financial or material support) for your study. List the grants or organizations that supported your study, including funding received from your institution. 
b) State what role the funders took in the study. If the funders had no role in your study, please state: “The funders had no role in study design, data collection and analysis, decision to publish, or preparation of the manuscript.”
c) If any authors received a salary from any of your funders, please state which authors and which funders.
d) If you did not receive any funding for this study, please state: “The authors received no specific funding for this work.”

Please include your amended statements within your cover letter; we will change the online submission form on your behalf.

This study received a small grant from IPA’s Peace and Recovery Program. The funders had no role in study design, data collection and analysis, decision to publish, or preparation of the manuscript. No authors received a salary from the funders.

This has been added to our updated cover letter.

5. We note that you have stated that you will provide repository information for your data at acceptance. Should your manuscript be accepted for publication, we will hold it until you provide the relevant accession numbers or DOIs necessary to access your data. If you wish to make changes to your Data Availability statement, please describe these changes in your cover letter and we will update your Data Availability statement to reflect the information you provide.

Here is the link:

<https://dataverse.harvard.edu/dataset.xhtml;jsessionid=438edef13da12805ee8f2a5d7a9d?persistentId=doi%3A10.7910%2FDVN%2FVO7SUO&version=&q=&fileTypeGroupFacet=&fileAccess=&fileSortField=date>

6. Please include captions for your Supporting Information files at the end of your manuscript, and update any in-text citations to match accordingly. Please see our Supporting Information guidelines for more information: <http://journals.plos.org/plosone/s/supporting-information>. 


[Note: HTML markup is below. Please do not edit.]

Reviewers' comments:

Reviewer's Responses to Questions

**Comments to the Author**

1. Is the manuscript technically sound, and do the data support the conclusions?

The manuscript must describe a technically sound piece of scientific research with data that supports the conclusions. Experiments must have been conducted rigorously, with appropriate controls, replication, and sample sizes. The conclusions must be drawn appropriately based on the data presented.

Reviewer #1: No

2. Has the statistical analysis been performed appropriately and rigorously?

Reviewer #1: I Don't Know

3. Have the authors made all data underlying the findings in their manuscript fully available?

The [PLOS Data policy](http://www.plosone.org/static/policies.action#sharing) requires authors to make all data underlying the findings described in their manuscript fully available without restriction, with rare exception (please refer to the Data Availability Statement in the manuscript PDF file). The data should be provided as part of the manuscript or its supporting information, or deposited to a public repository. For example, in addition to summary statistics, the data points behind means, medians and variance measures should be available. If there are restrictions on publicly sharing data—e.g. participant privacy or use of data from a third party—those must be specified.

Reviewer #1: Yes

4. Is the manuscript presented in an intelligible fashion and written in standard English?

PLOS ONE does not copyedit accepted manuscripts, so the language in submitted articles must be clear, correct, and unambiguous. Any typographical or grammatical errors should be corrected at revision, so please note any specific errors here.

Reviewer #1: No

5. Review Comments to the Author

Please use the space provided to explain your answers to the questions above. You may also include additional comments for the author, including concerns about dual publication, research ethics, or publication ethics. (Please upload your review as an attachment if it exceeds 20,000 characters)

Reviewer #1: This manuscript reports on a text message intervention implemented in Kenya. The study tested the effect of text messaging on message recall, attitudes, intentions, and stigma related to COVID-19.

Include the hypothesis in the manuscript abstract to clarify the dependent variable(s).

We have added the hypothesis to the abstract.

Background

Paragraph 1
- Review sentence starting with, “Initially, all…”

Thank you for catching this we have revised the sentence.

- Ideally, the measures employed/ implemented (by Kenyan MoH) should be referenced.

We have added some text to describe the initial MoH response.

Paragraph 2
- It may be worth clarifying what the significant toll was.

We have added that this refers to the mental health impacts of Ebola.

- Clarify the last sentence; did C19 messaging cause community to socially isolate people with SC2, and also stigmatize healthcare workers?
edited for clarity.

Paragraph 3
- The effect of text messaging to influence health behaviour is variable and it may be instructive for the reader to understand this going into the methods section. There are a number of systematic reviews on text messages and health behavior that may be worth covering in the introduction.
we have added some more information here including a meta analysis, and we cite multiple papers on text messaging and behavior change. Overall we tried to keep the introduction short but have added to make sure the literature is clearly stated.

Additionally, provide evidence for the hypotheses that will be tested – ie the effect of the independent variable(s) (text message content) upon the dependent variables 1) recall, 2) attitudes, 3) intentions 4) stigma.

We wrote the following sentence to describe our research question Our primary research question was: Can we improve knowledge, attitudes (stigma, specifically), and behavioral intentions related to the isolation of cases/caregiving for those infected with COVID-19 using a brief text-message-based intervention promoting individual or social motives?

And have now added “We hypothesized that text messages tailored with either social or personal benefit framing would improve the recall, attitudes, intentions, and perceptions of stigma regarding personal isolation if infected and of caring for a sick family member at home, in adherence with local guidelines at the time”

Methods
- Data analysis – state what the measures were, and how the outcome was measured. How were knowledge, attitudes, and intentions measured? Was there a hypothesis? What was the hypothesis? The hypothesis seems inferred; that texts containing personal and social motivations will increase knowledge, change attitudes, and increase intentions to care for household contacts.

As noted above we added this sentence to the end of the background section: “We hypothesized that text messages tailored with either social or personal benefit framing would improve the recall, attitudes, intentions, and perceptions of stigma regarding personal isolation if infected and of caring for a sick family member at home, in adherence with local guidelines at the time”

Regarding measures, we have a short section called “study outcomes” on page 6. We have added to clarify that these were measured through responses to our mobile phone survey.

- It may be worth clarifying the hypothesis/ hypotheses, reflecting back on the evidence/ literature provided in the introduction.

We have added some information in the methods.

Discussion
- The “no control condition” is a major limitation on the study. It may be worth explaining the impact on the outcome variables. What is the extent of the influence of non-study text messages, and other information on the dependent variables, recall, attitudes, intentions, stigma? To what extent is it possible, considering this limitation, to draw conclusions and make inferences about the intervention (independent variable)?

We have added in the discussion that since EVERYONE in Nairobi was receiving text messages we do not have a pure control group. However, our aim was to measure the added benefit of a social or personal benefit framing of the messages, not the impact of any message. So this is the main difference – we were exploring how the tailoring of the message had an impact vs the receipt of any message. This was the best possible under these conditions which were the early days of the pandemic (pre vaccine, etc) when there was a lot of confusion and a lot of stigma. We worked with the Government of Kenya to design and test these messages to inform their information campaign when there was little known about COVID-19.

There are a few typographical/ sentence structure errors that can easily be addressed.

Thank you, we have re-read the manuscript and checked for grammatical and typographical errors.

6. PLOS authors have the option to publish the peer review history of their article ([what does this mean?](https://journals.plos.org/plosone/s/editorial-and-peer-review-process#loc-peer-review-history)). If published, this will include your full peer review and any attached files.


If you choose “no”, your identity will remain anonymous but your review may still be made public.


**Do you want your identity to be public for this peer review?** For information about this choice, including consent withdrawal, please see our [Privacy Policy](https://www.plos.org/privacy-policy).

Reviewer #1: **Yes:** Philip Smith
