# Supplementary material for: The limits of nudging: Results of a randomized trial of text messages to promote home-based caregiving and reduce perceptions of stigma for COVID-19 patients in Kenyan informal settlements
Source: PLoS One. 2024 Aug 29;19(8):e0305206. doi: 10.1371/journal.pone.0305206 (PMC11361586; doi:10.1371/journal.pone.0305206)
Supplement: Attachment — Submitted filename: response_to_reviewers.docx [file pone.0305206.s005.docx]

PONE-D-23-11319R1

The limits of nudging: Results of a randomized trial of text messages to promote home-based caregiving and reduce perceptions of stigma for COVID-19 patients in Kenyan informal settlements

**ACADEMIC EDITOR:** Thank you for your patience as we struggled to find reviewers over the holiday period.

This article has merit, but requires additional content and restructuring to ensure that readers can follow the story and also that there is enough detail to enable replication or adaptation with future designs. With regards to adding an additional table by one review, I do not require this, though you may want to accept this suggestion.

 Some specifics I will be looking for in the revised manuscript:

- As noted by both reviewers, ensure that the title aligns with the content, in particular the term 'nudge.' Preferably add further detail about your definition(s) of nudging in the body of the manuscript.

We have clarified but nudging simply refers to using information to shift a behavior and is commonly used in development economics and related studies. We have added text in the background and discussion to clarify how we are using the term.

If this is not sufficient we can remove the first part of the title.

- To address reviewer questions about the theoretical basis for selection of private and social benefit frames, at a minimum include references in the sentence that begins 'Based on existing literature' on p.4 [NB: references 17-20 that were cited on p.5 seem relevant here]

We have added these references here, since these were used to inform the theoretical basis for the selection of each of the message frames.

- Clarify what is meant by informed in the sentence 'sent informed participants' on p.4. Consider rewording

Thank you, this appears to be an error we have corrected. The control condition was just information.

- While I assume English, specify the language of messages in the behavioral intervention section (p.4-5) and address literacy rates of the sample (if known) in the section on sample (p.5)

These were in both English and Swahili, we have added this (top of page 6).

- Consider reframing the title for Table 3. The statements present behavioral expectations vs attitudes towards stigma (p.9)

The Table title has been edited.

- Ensure the basis for the denominators for Tables 4-6 are clear in the text, to ensure accurate interpretation. For instance, are the numbers lower in Table 4 because only those who recalled messages from the Population Council/Mpayer (see Table 2) were included?

We have added the denominators for clarity into each table. We have also removed table 5, it was confusing as written and did not add to the findings.

- Specify which messages in Tables 4-6 belong to control/all vs the private or social benefit arms. This can be done next to the statements in the first column.

The column names in table 4 are the type of message received (control group, private benefit group, and social benefit group).

In Table 5, the columns are comparing results for intention to treat analysis, vs per protocol analysis, and the message groups are in the rows because the personal benefit group and social benefit group are being compared to the control group, adjusting for other variables.

- Given the study design (Figure 1), where private and social benefit messages were only sent to one arm, how does the research team explain recall of the same messages among both control and other arms as presented in Tables 4-5? This needs to be unpacked more in discussion. Similarly the fact that only 2 messages were sent to each group but they recalled more messages needs to be addressed overtly in the discussion.

We have now dropped table 5, but it was meant to show that people were receiving many messages from many sources early in the pandemic, and it was confusing/people were overwhelmed with information, not all of which was accurate. So people received 2 from our project, but also received a variety of other messages. Since we have dropped table 5 this is less confusing but we have still added a sentence to the discussion to refer to this information overload.

In our RCT, we randomly assigned people to receive one of three versions of the SAME message. So it makes sense they all would recall similar information the difference was the perception and if they took up the behavior based on the framing of that message.

- Further to a reviewers request for more clarity on Table 5, move the current column 1 heading above the other 4 columns and create new headings for both the table and first column that reflects content

We removed table 5.

- In the discussion, paragraph 2, p.13, ensure that your study results are discussed in the context of gauging behavioral intention and not actual behavior.

A sentence has been added to reflect we are only able to collect behavioral intention not actual behavior.

- The discussion should also expand on findings about structural determinants (wealth and space) on intention to care (Table 6 results) as part of the 'other kinds of interventions' mentioned at the bottom of p.13

We have added two new references and discuss wealth and space in relation to intention to care.

Reviewers' comments:

Reviewer's Responses to Questions

6. Review Comments to the Author

Reviewer #2: This research makes an important contribution towards understanding the limitations of using SMS to drive behaviour change. However, the paper could be improved in some areas to make it clearer to practitioners how they should go about intervention design through SMS and what the benefits and limitations are. It would be good to get a brief overview of the literature and what we have learnt about nudging. It was also difficult to understand how the team went about designing their own intervention. I have attached comments related to specific sections in the paper, and here is a summary:

- The title is making a large statement about nudging. It would be good to get some background information in the introduction section about nudging. What constitutes a nudge? Under what circumstances is it supposed to work, and under which is it not supposed to from a theoretical perspective? What are the merits and what are the limitations?
The term ‘nudging’ is commonly used in behavioral economics and behavioral sciences to refer to a subtle intervention, like an SMS, that aims to shift a behavior and help people make a decision. We can remove the first part of the title, but we feel that throughout the background and discussion we describe the term and what we mean by it here. The incentive or ‘nudge’ here is the SMS. While a very simple intervention, we wanted to ensure we were using the same platform as the Government of Kenya (they were only using SMS and some radio/tv information at the time) so that if effective, they could carry on the SMS campaign. Our hypothesis, as stated in the paper, is that the way the SMS are framed could make them more or less likely to be recalled and more or less likely that the information be used.

- It would be good to have a clearer understanding of the theoretical basis that the team used to inform the design of their SMS and why specific behavioural determinants were focused on.
At the time of the study, COVID-19 had just spread to Kenya. We worked with the Government of Kenya (GoK) to discuss types of messages and approaches they might use. The GoK was planning to send SMS which is why we restricted our study to this very basic form of communication (vs creating a website or sending WhatsApp messages – we wanted to align with the government plan). At the time of this study, the guidance was to quarantine for 10 days which meant a reliance on family members for food and medical care.

In the background section we clarify that we used the same approach as the Government of Kenya to ensure the opportunity for scale up and the rationale.

- In the discussion it would be good to get some insights on whether the messenger effect is at play: so would the same messages be more effective if they came from another source, like a WhatsApp group?
Unfortunately we only used SMS for this study, because we wanted to keep to the same channel that the government of Kenya would be implementing if this was scaled up. We cant speak to whether other channels or approaches would be more effective since we only tested this one, but it’s a really interesting question for future study!

- Consider adding some information on alternative behaviour change techniques that could be used through other modalities that may be more effective from a theoretical perspective.
We were restricted in our approach, in part because it was during the lockdown phase of the pandemic so we could not do anything in person, and also because we wanted to keep to the approaches and channels that would be feasible for the Government of Kenya. We discussed with them, and they were using text/SMS only. While we agree there may be potential for other approaches, since we were not able to explore them, we did not include very much on other modalities since they were not available to us.

- How would the others suggest that governments go about public health mass media communication in an effective way? What has worked better in other countries?
We include a few examples in the background section paragraph 4, from the US, Denmark and a meta-analysis from multiple sites, related to messaging during the pandemic.

Here are the comments per page:
Page 10: Could you give a clearer theoretical background to the intervention design? Could you be a bit clearer on why these behavioural determinants were chosen? What theories informed your intervention? Were different phrasings pre-tested before this was chosen? Were the messages translated?
At the time of the study, the gok was trying to get people to adhere to the 10 day quarantine period, which required family members to take care of infected people, bringing them food/supplies, etc. At the time of the study there was no COVID vaccine, and no real treatments, so quarantine was the best option. We discussed with the GoK what modality they would be using for messaging, and were told to use SMS messages. This is why we did not test other types of messaging campaign – we wanted to ensure that we used the same approach and could scale up if our results were positive.

The messages were translated and sent out in English and Swahili to ensure everyone could understand them. We did some basic piloting for comprehension, but mostly designed the messages in collaboration with the GoK.

We randomized SMS messages because this is how the GoK was sending out information. We randomized the messages to ensure the study was rigorous as this is the most rigorous study design.

Page 11: Can you add a table summarizing some of the information that led to the SMS phrasing decisions? Please explain how the surveys administered. Were the survey questions for attitudes and intentions based on existing scales or guidance? If so, which ones?
In the methods section, specifically page 4-5, we outline that the surveys were conducted on the phone before and after we sent the SMS messages out. The phrasing was simply based on conversations with the GoK to ensure that we reflected what they were planning to communicate at this early time during the pandemic when quarantining was required. At the time there were no existing questions or scales to use since the pandemic was in its early days.

We do not think adding a table would be particularly useful since the information is already laid out on page 5, where we have a short paragraph for each condition and why it was selected.

Page 17: The information on Table 5 is not clear, could you present it differently?

We have removed table 5.

Reviewer #3: Thank you for the opportunity to review this paper. Exploring the use of tailored messaging to improve outcomes in a cost-effective way is a pertinent topic and the title is good.

The article could benefit from a better structured and written background. Please check grammar and write for clarity e.g. adding years to dates. It should also follow a more coherent “story” or focus e.g. text messages are a cheap intervention which has potential for large reach but unknown if tailored messaging is better than plain informational messaging... Please link to COM-B model if use this in discussion. The hypothesis is clear.
We have reviewed the background, all dates are complete, and we have aimed to clarify and streamline language. We re-ordered the tables slightly to follow the ‘story’ and have removed table 5. We also outlined that the SMS approach was because these were used by the GoK and they are cost effective, simple and have wide reach, the first sentence of the conclusion is “Text messages are inexpensive and may rapidly and easily reach large numbers of people in emergency settings.”.

More information is needed on the measures in the methods section, especially in describing outcomes and how recall of the PC-specific (vs government and other) messages was measured. It is unclear who, in the households, was interviewed - the phone owner? There were IDs but how was this ensured that it was the same person? What are the implications of using pre-identified HH with adolescents vs general community? What about exposure to other messaging beyond text e.g. mass media? Was this not measured? The last sentence of the existing measures section should probably be in limitations.

In the study outcomes paragraph, we write that these were assessed through responses to our survey questions, and we have added “collected via phone call”. We asked questions about recall and receiving messages, and rely on self-report from the respondents. We have added to the limitations that we rely on self-report.

We have added that the same person was interviewed each time.

We have removed table 5 which described the other messaging people received, and added a sentence in the discussion regarding the overwhelming amount of information (and misinformation) spreading during the early days of the pandemic.

we have added to the limitations a sentence regarding the implications of using pre-identified households from our other survey.

In the discussion, please link back to the background more (with a clearer "story") and to the framework used. Is the conclusion about not being amenable right? Should it not be about needing to explore whether messaging needs to be either accompanied by practical information on how to address those contextual factors or a structural intervention such as a grant?

we have added some text to the discussion section including the need to address contextual factors like poverty and housing infrastructure to be effective.

The title is nice but there is limited use of the term "nudge" in ther article itself.
The term nudging is referred to throughout the background and discussion, and we have added even more information about nudging approaches and theory, hopefully this makes the title more clear. We are also happy to remove this term from the title for publication.
